# Plasma, salivary and urinary cortisol levels following physiological and stress doses of hydrocortisone in normal volunteers

**DOI:** 10.1186/1472-6823-14-91

**Published:** 2014-11-26

**Authors:** Caroline Jung, Santo Greco, Hanh HT Nguyen, Jui T Ho, John G Lewis, David J Torpy, Warrick J Inder

**Affiliations:** Department of Endocrinology and Diabetes, St Vincent’s Hospital, Melbourne, VIC Australia; School of Medicine, The University of Melbourne, Melbourne, VIC Australia; Department of Biochemistry, Melbourne Pathology, Melbourne, VIC Australia; Endocrine and Metabolic Unit, Royal Adelaide Hospital, Adelaide, South Australia Australia; Steroid & Immunobiochemistry Laboratory, Canterbury Health Laboratories, Christchurch, New Zealand; Endocrine Research, Hanson Institute, Adelaide, South Australia Australia; School of Medicine, The University of Adelaide, Adelaide, South Australia Australia; Department of Diabetes and Endocrinology, Princess Alexandra Hospital, Brisbane, QLD Australia; School of Medicine, The University of Queensland, Brisbane, QLD Australia

**Keywords:** Adrenal Cortex, HPA axis (hypothalamic-pituitary-adrenal), Cortisol, Hydrocortisone

## Abstract

**Background:**

Glucocorticoid replacement is essential in patients with primary and secondary adrenal insufficiency, but many patients remain on higher than recommended dose regimens. There is no uniformly accepted method to monitor the dose in individual patients. We have compared cortisol concentrations in plasma, saliva and urine achieved following “physiological” and “stress” doses of hydrocortisone as potential methods for monitoring glucocorticoid replacement.

**Methods:**

Cortisol profiles were measured in plasma, saliva and urine following “physiological” (20 mg oral) or “stress” (50 mg intravenous) doses of hydrocortisone in dexamethasone-suppressed healthy subjects (8 in each group), compared to endogenous cortisol levels (12 subjects). Total plasma cortisol was measured half-hourly, and salivary cortisol and urinary cortisol:creatinine ratio were measured hourly from time 0 (between 0830 and 0900) to 5 h. Endogenous plasma corticosteroid-binding globulin (CBG) levels were measured at time 0 and 5 h, and hourly from time 0 to 5 h following administration of oral or intravenous hydrocortisone. Plasma free cortisol was calculated using Coolens’ equation.

**Results:**

Plasma, salivary and urine cortisol at 2 h after oral hydrocortisone gave a good indication of peak cortisol concentrations, which were uniformly supraphysiological. Intravenous hydrocortisone administration achieved very high 30 minute cortisol concentrations. Total plasma cortisol correlated significantly with both saliva and urine cortisol after oral and intravenous hydrocortisone (*P* <0.0001, correlation coefficient between 0.61 and 0.94). There was no difference in CBG levels across the sampling period.

**Conclusions:**

An oral dose of hydrocortisone 20 mg is supraphysiological for routine maintenance, while stress doses above 50 mg 6-hourly would rarely be necessary in managing acute illness. Salivary cortisol and urinary cortisol:creatinine ratio may provide useful alternatives to plasma cortisol measurements to monitor replacement doses in hypoadrenal patients.

## Background

Hydrocortisone is the standard therapy for patients with adrenal insufficiency [1]. The traditional hydrocortisone replacement regimen had been 20 mg in the morning and 10 mg later in the day [2], based on earlier estimates of endogenous cortisol production of 12–15 mg/m^2^/day [3]. However, more recent studies showed that the mean cortisol production in normal subjects may be as low as 5.7 mg/m^2^/day [4, 5], or 9–11 mg/m^2^/day [6], leading to recommendations of a total daily replacement dose of 15–20 mg hydrocortisone [7, 8]. Despite these recommendations, higher doses are still used widely in clinical practice with glucocorticoid replacement equivalent to hydrocortisone doses of ≥30 mg daily being prescribed in 67% [9] and 91% [10] of outpatients with adrenal insufficiency. This is of considerable importance given the association with hydrocortisone doses above 25–30 mg/day and increased mortality [9, 11].

More physiological circadian glucocorticoid therapy can be achieved by using continuous intravenous [12] or subcutaneous hydrocortisone infusion [13], delayed-release [14, 15] or dual-release [16] oral hydrocortisone preparations. Recently, delivery of pulsatile sub-cutaneous hydrocortisone in an attempt to mimic ultradian pulsatility has been reported [17]. However, none of these treatment regimens can take into consideration the variation in end-organ glucocorticoid sensitivity which cannot be measured biochemically [1, 18], and currently the majority of patients remain on traditional twice or thrice daily hydrocortisone regimens [19].

There is considerable inter-individual variability in the cortisol profiles after the administration of glucocorticoids [20, 21]. However, there is no consensus on how to determine the most appropriate replacement dose for individual patients [1]. Some adjust by clinical features [10, 22], whereas others have advocated the use of plasma cortisol day curves [2, 8, 20, 23, 24]. An algorithm has been proposed [21], using the plasma cortisol concentration 4 hours after the morning hydrocortisone dose to help guide dose adjustment, but there are no data as to how frequently this is used by clinicians or the effect on clinical outcome.

Previous studies on plasma cortisol day curves have measured total cortisol which includes cortisol bound to corticosteroid-binding globulin (CBG) and free (unbound) cortisol. Plasma free cortisol is technically difficult to measure and not widely available, but can be calculated using a validated formula [25]. A measure of free cortisol can also be estimated using saliva and urine samples. While some studies have concluded that monitoring salivary cortisol does not add significantly to the evaluation of adequate glucocorticoid replacement therapy [1, 18, 26], another study found the correlation between paired plasma and salivary cortisol concentrations was far better [27]. The use of 24 h urinary free cortisol (UFC) has been advocated for monitoring of overall glucocorticoid replacement quality [28, 29]. However, 24 h UFC has limitations, as it does not account for fluctuations in cortisol concentrations throughout the day or the peaks and troughs following dosing [1, 18]. There are no data on cortisol day curves using spot urine measurements.

Patients with adrenal insufficiency require additional glucocorticoid doses during surgery or medical illness, but there is no universally accepted regimen for glucocorticoid supplementation therapy [30]. Recommendations regarding glucocorticoid coverage during stress are largely based on expert opinion, and there are few published data on cortisol levels reached in the plasma, saliva and urine following intravenous hydrocortisone given at “stress’” doses.

The main aim of the study was to investigate whether noninvasive methods of measuring cortisol in the saliva or urine provide useful information in assessing individual hydrocortisone dose requirements. Secondary aims included the assessment of cortisol levels in plasma, saliva and urine following both physiological and stress hydrocortisone dosing, and the inter-individual variability in cortisol pharmacokinetics.

## Methods

### Subjects

Twelve healthy participants were recruited for the study. All completed the baseline study on day 1 to measure endogenous cortisol levels (control day). Of the 12 participants, four then participated in the oral hydrocortisone study (day 2), four participated in the intravenous hydrocortisone study (day 2), and four participated in both studies on separate days (days 2 and 3). Therefore, there were eight participants each in the oral and intravenous hydrocortisone groups. The clinical characteristics are shown in Table 1. There were no significant differences in the age (*P* = 0.92), weight (*P* = 0.96) or BMI (*P* = 0.68) between the groups receiving oral versus intravenous hydrocortisone. Exclusion criteria were pre-existing Cushing’s syndrome, adrenal insufficiency, renal impairment, psychiatric disorders, alcoholism, pregnancy, and the use of glucocorticoid medication, oestrogen or drugs known to affect the metabolism of hydrocortisone [31] or dexamethasone [32].

**Table 1:** **Clinical characteristics of subjects**

	Endogenous cortisol	Oral hydrocortisone group	Intravenous hydrocortisone group
	Day 1		
Number of subjects [M:F]	12 [3:9]	8 [1:7]	8 [3:5]
Age (years)^1^	44.7 ± 4.6	46.6 ± 5.5	43.4 ± 5.8
	(19–59)	(21–59)	(19–58)
Weight (kg)^1^	67.0 ± 4.1	68.7 ± 5.5	66.9 ± 5.6
	(50–101)	(50–101)	(55–101)
BMI (kg/m^2^)^1^	25.3 ± 1.5	26.8 ± 2.1	24.4 ± 1.9
	(20–37)	(20–37)	(21–37)

M - male; F - female; BMI - body mass index.

^1^Data are presented as mean ± SEM (range).

### Study design

On day 1 (endogenous control day), plasma samples were collected half-hourly, and saliva and urine samples were collected hourly from 0 (between 0830 and 0900) to 5 h. On day 2, the first plasma, saliva and urine samples were collected at time 0 (between 0830 and 0900) immediately before the administration of oral hydrocortisone 20 mg (Hysone^®^, Alphapharm, Millers Point, Australia) or intravenous bolus of hydrocortisone 50 mg (Solu-Cortef^®^, Pfizer Australia, West Ryde, Australia). After the administration of hydrocortisone, plasma samples were collected half-hourly, and saliva and urine samples were collected hourly to 5 h. For subjects who participated in both oral and intravenous studies, days 2 and 3 were separated by more than 4 weeks. The collection time points on day 3 were same as on day 2. Endogenous cortisol production was suppressed by oral administration of 4 mg of dexamethasone at 2300 h on the night before day 2 and 3. The dose of 4 mg was used to ensure that endogenous ACTH and cortisol remained suppressed throughout the following day.

Participants were instructed to have their usual breakfast at home prior to attending and lunch was taken between 3.5 h and 5 h. Before the collection of saliva samples, subjects were asked to rinse their mouth to remove any residual food (all groups) or residual hydrocortisone medication (oral hydrocortisone group) in the oral cavity. Saliva samples were collected by drooling into a 5 mL plastic tube using a straw.

### Assays

Plasma cortisol was measured by a commercial immunoassay (Modular Analytics E170, Roche Diagnostics, Mannheim, Germany). The analytical sensitivity was 8.5 nmol/L. The intra-assay coefficient of variation was 1.7% for cortisol at 129 nmol/L. The inter-assay coefficient of variation was 4.7% for cortisol at 102 nmol/L and 2% for cortisol at 940 nmol/L. Salivary cortisol was measured by a commercial immunoassay (Immulite 2000, Siemens Healthcare Diagnostics, Deerfield, IL, USA) after extraction with ethyl acetate. The analytical sensitivity was 3 nmol/L. The inter-assay coefficient of variation was 13.8% for cortisol at 3.9 nmol/L, and 14.3% for cortisol at 7.4 nmol/L. Urinary cortisol:creatinine ratio was calculated by dividing urinary cortisol concentration by urinary creatinine. Urinary cortisol concentration was measured by a commercial immunoassay (Immulite, 2000, Siemens Healthcare Diagnostics, Deerfield, IL, USA) after extraction with ethyl acetate. The analytical sensitivity was 27 nmol/L. The inter-assay coefficient of variation was 13.1% for cortisol at 38 nmol/L, and 7.5% for cortisol at 323 nmol/L. The cross reactivity with cortisone was 0.3% for both urinary and salivary cortisol. Urinary creatinine was measured by Roche Cobas Integra 800 creatinine Jaffe assay (Roche Diagnostics, Mannheim, Germany). The analytical sensitivity was 0.027 mmol/L. The intra- and inter-assay coefficient of variation was 1.4% and 2.5%, respectively, for creatinine at 2.16 mmol/L. Plasma free cortisol was calculated using Coolens’ equation [25].

### Data analysis and statistics

Individual and mean cortisol levels were plotted against time. For statistical purposes, the value corresponding to the limit of detection of assays was used for undetectable concentrations. Cortisol concentrations after oral hydrocortisone were compared with the endogenous cortisol data on day 1. Cortisol concentrations after intravenous hydrocortisone were compared with the oral hydrocortisone group. ANOVA was used for the comparison across time points with Bonferroni correction for multiple testing performed to determine statistical significance for each time point. CBG levels were compared at baseline (time 0) on the control day and post-dexamethasone using the paired *t*-test. For the 4 participants who received both iv and oral hydrocortisone (and therefore received dexamethasone twice), the mean of the two concentrations was used in the analysis, along with the single values for the remaining 8 participants. The *t*-test was used for comparison between two groups of data. Statistical significance was taken as *P* <0.05.

The peak level and time to peak level (T_max_) were determined using actual collection time points. Area under cortisol level-time curve from 0 to 5 h (AUC_0–5_), were determined using the trapezoid method. Variability in peak cortisol and AUC_0–5_ was expressed by the coefficient of variation (CV) which was calculated by the following equation: CV (%) = (standard deviation/mean) × 100. Total body clearance was calculated from dose/AUC_0-infinity_.

Correlation between plasma and salivary cortisol or urinary cortisol:creatinine ratio on day 1 (endogenous cortisol levels) were determined by using cortisol measurements at times 0, 1 h, 2 h, 3 h, 4 h and 5 h. Correlation between plasma and salivary cortisol or urinary cortisol:creatinine ratio after the administration of oral or intravenous hydrocortisone was determined by using cortisol measurements at 1 h, 2 h, 3 h, 4 h and 5 h; cortisol levels at time 0 were not used because of exogenous suppression by dexamethasone. The Pearson correlation coefficient was represented by *R*.

Statistical analyses were performed using Prism version 4.0 (GraphPad, San Diego, CA, USA). Pharmacokinetic analyses were performed using PK Functions for Microsoft Excel software program (Joel Usansky, Atul Desai and Diane Tang-Liu, Department of Pharmacokinetics and Drug Metabolism, CA, USA).

### Ethics approval

The study protocol was approved by the St. Vincent’s Hospital (Melbourne, Australia) Human Research Ethics Committee. Written informed consent was obtained from each participant after full explanation of the purpose and nature of all procedures used.

## Results

### Control day

**Figure 1:**
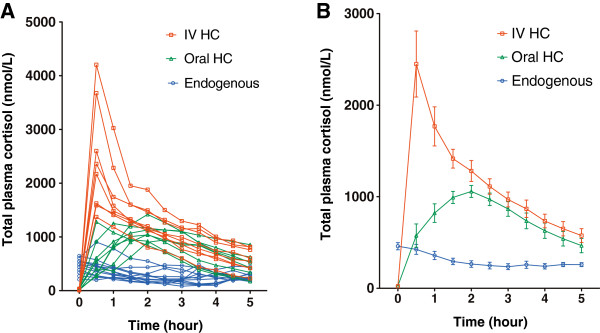
**Total plasma cortisol concentrations after oral and intravenous hydrocortisone.** Individual **(A)** and mean (± SEM) **(B)** total plasma cortisol levels: endogenous (blue circle) levels and after oral (green triangle) or intravenous (red square) hydrocortisone.

The pattern of endogenous cortisol measurements was consistent with the known diurnal rhythm (Figure 1). There was high variability in endogenous peak cortisol and AUC_0–5_ values (Table 2). There was no difference in mean endogenous CBG concentrations at time 0 and 5 h (*P* = 0.5)

**Table 2:** **Pharmacokinetic parameters of cortisol following oral or intravenous administration of hydrocortisone (HU)**

		Day 1	Oral HC	IV HC
Plasma cortisol	Peak^1^	505 ± 46	1135 ± 60	2450 ± 361
	(nmol/L)	(318–905)	(943–1419)	(1372–4205)
	Peak CV^2^	31%	15%	42%
	T_max_^1^		1.7 ± 0.2	0.5 ± 0
	(h)		(0.5-2 5)	
	AUC_0-5_^1^	1469 ± 195	3720 ± 280	5775 593
	(nmol/L.h)	(963–2433)	(2615–4663)	(3458–8684)
	AUC_0–5_ CV^2^	37%	21%	29%
Plasma free cortisol	Peak^1^	64 ± 12	179 ± 20	694 ± 112
	(nmol/L)			
	Peak CV^2^	63%	31%	50%
	T_max_^1^		1.7 ± 0.2	0.5 ± 0
	(h)			
	AUC_0-5_^1^	145 ± 23	462 ± 67	1236 ± 170
	(nmol/L.h)			
	AUC_0-5_CV^2^	54%	41%	39%
Salivary cortisol	Peak^1^ (nmol/L)	19 ± 4	100 ± 16	348 ± 88
		(6–48)	(58–164)^3^	(106–816)
	Peak CV^2^	80%	40%	71%
	T_max_^1^		1.7 ± 0.2	1 ± 0
	(h)		(1–2)^3^	
	AUC_0-5_^1^	38 ± 4	241 ± 30	610 ± 147
	(nmol/L.h)	(21–68)	(142–325)^3^	(210–1422)
	AUC_0–5_ CV^2^	40%	31%^3^o	68%
Urinary cortisol: creatinine ratio	Peak^1^	73 ± 18	789 ± 164	4635 ± 926
	(nmol: mmol)	(29–244)	(227–1468)	(480–8192)
	Peak CV^2^	88%	59%	57%
	T_max_^1^		2.1 ± 0.3	1.1 ± 0.1 (1—2)
	(h)		(1–3)	
	AUC_0-5_^1^	216 ± 41	1732 ± 316	7280 ± 1488
	(nmol:mmol.h)	(93–558)	(440–2839)	(1058–10715)
	AUC_0–5_ CV^2^	65%	51%	58%

^1^Data are presented as mean ± SEM (range). T_max_ is the time to reach peak concentration.

^2^Variability in peak cortisol and AUC_0–5_ values are expressed by coefficients of variation (CV).

^3^n = 6 (salivary cortisol data from two subjects were excluded because of extreme salivary cortisol outliers at 1 h, suggesting oral contamination).

### Dexamethasone suppression

Suppression of the endogenous hypothalamic-pituitary-adrenal axis by dexamethasone was confirmed in all subjects by the measurement of cortisol levels at time 0 before the administration of oral or intravenous hydrocortisone. At time 0, total plasma cortisol was less than 50 nmol/L and salivary cortisol was less than 3 nmol/L. Dexamethasone had no significant effect on CBG concentrations, mean baseline CBG on control day 583 ± 55 nmol/l compared to mean baseline CBG post-dexamethasone 666 ± 41 nmol/L (*P* = 0.24).

### Oral administration of hydrocortisone

**Figure 2:**
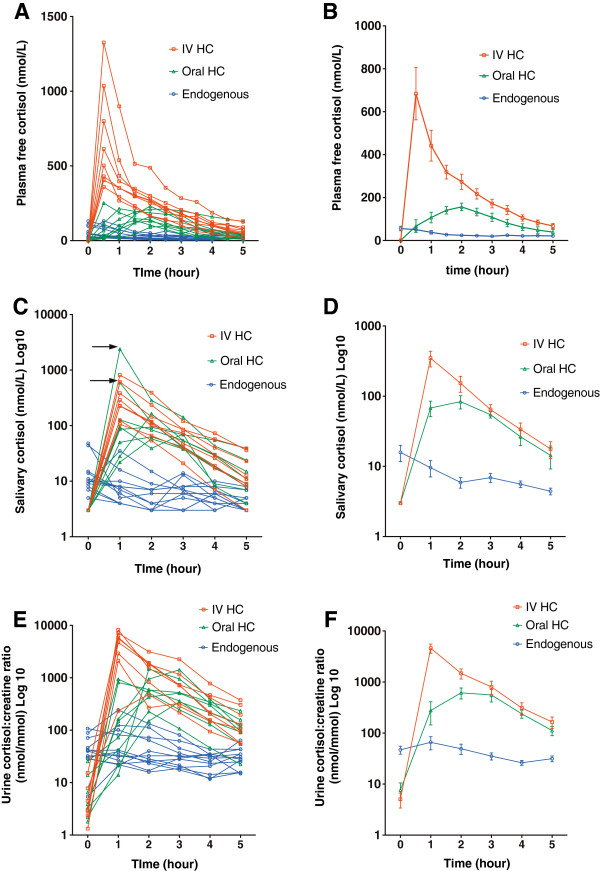
**Plasma free, salivary and urine cortisol concentrations after oral and intravenous hydrocortisone.** Individual free cortisol levels in the plasma **(A)**, saliva **(C)** and urine **(E)**, and mean (± SEM) free cortisol levels in the plasma **(B)**, saliva **(D)** and urine **(F)**: endogenous (blue circle) levels and after oral (green triangle) or intravenous (red square) hydrocortisone. The arrows in Figure 2 **C** indicate two extreme salivary cortisol outliers 1 h after oral hydrocortisone (596 and 2385 nmol/L).

After the administration of oral hydrocortisone 20 mg, there was no change in CBG concentrations between time 0 and 5 h (*P* = 0.97 by ANOVA). Total plasma cortisol concentrations were higher than endogenous data (*P* <0.0001 by ANOVA, Figure 1B), and the *post hoc* analysis showed that the differences were statistically significant (*P* <0.05) at each of the time points from 1 h to 4.5 h. Similar results were obtained with calculated plasma free cortisol data (*P* = 0.0001 by ANOVA, Figure 2B).

Salivary cortisol concentrations increased within 2 hours after ingestion of oral hydrocortisone (Figure 2C). Two subjects (25%) had very high salivary concentrations of 596 nmol/L and 2385 nmol/L measured at 1 h after oral hydrocortisone (indicated by arrows in Figure 2C), compared to peak salivary cortisol concentrations in other six subjects (range, 58–164 nmol/L), suggesting contamination by the residual hydrocortisone. Therefore, salivary cortisol data from these two subjects were excluded from subsequent analyses. Salivary cortisol concentrations were higher than endogenous data (*P* <0.0001 by ANOVA, Figure 2D), and the differences were statistically significant from 1 h to 3 h. Urinary cortisol:creatinine ratios were much higher than the endogenous data (*P* <0.0001 by ANOVA, Figure 2F), and the differences were statistically significant at 2 h and 3 h. The mean peak values in total and free plasma, salivary and urinary cortisol were 2-fold, 3-fold, 5-fold and 10-fold elevated, respectively, compared to the endogenous data (*P* = 0.00001, *P* = 0.00004, *P* = 0.0002 and *P* = 0.001, respectively, Table 2). There was considerable inter-individual variability in the peak cortisol and AUC_0–5_ values after oral hydrocortisone, but CV values were lower than the endogenous data (Table 2).

### Intravenous administration of hydrocortisone

There was no change in CBG concentrations between time 0 and 5 h after intravenous hydrocortisone (*P* = 0.99 by ANOVA). The intravenous bolus of hydrocortisone 50 mg achieved very high 30 minute cortisol levels (Figures 1A, and 2A, C, E). Wide inter-individual variability in the maximum cortisol and AUC_0–5_ values after intravenous hydrocortisone was observed (Table 2). Total plasma cortisol concentrations at 5 h were between 400–900 nmol/L in seven subjects (88%) and less than 400 nmol/L in one of eight subjects (12%). The mean (± SEM) clearance of intravenous hydrocortisone in the plasma was 20 ± 3 L/h, range, 13–34, compared to oral hydrocortisone (11 ± 2 L/h, range, 4–19, *P* = 0.013). By 2 h on the *post hoc* analysis, the differences in free cortisol measures (plasma, saliva, urine) after intravenous versus oral hydrocortisone were no longer statistically significant (Figure 2B, D, F).

### Correlation between plasma and salivary or urinary cortisol

**Figure 3:**
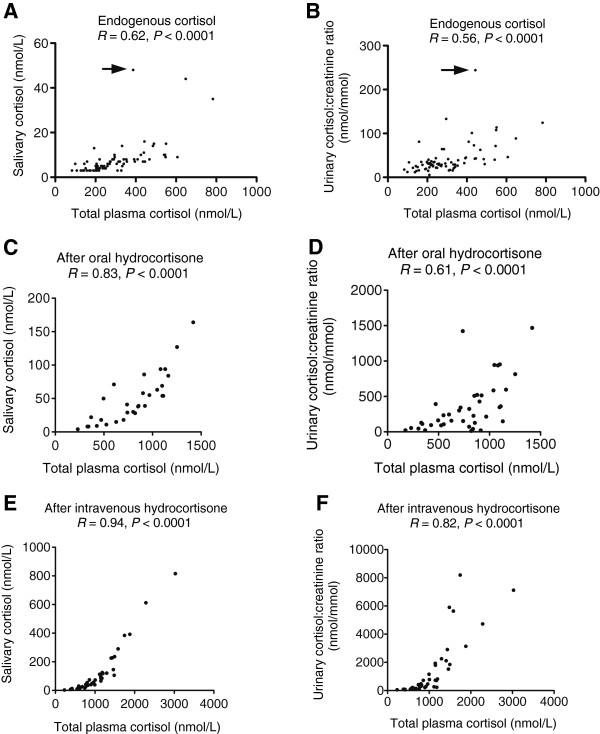
**Correlations between plasma, salivary and urine cortisol concentrations.** Correlation between endogenous plasma and salivary cortisol **(A)** and endogenous plasma cortisol and urinary cortisol:creatinine ratio **(B)**. The arrows indicate outlier results. Correlation between plasma and salivary cortisol **(C)** and plasma cortisol and urinary cortisol:creatinine ratio **(D)** in subjects given oral hydrocortisone. Correlation between plasma and salivary cortisol **(E)** and plasma cortisol and urinary cortisol:crearinine ratio **(F)** in subjects given intravenous hydrocortisone.

The relationship between endogenous total plasma and salivary cortisol concentrations (*R* = 0.62, *P* <0.0001) is shown in Figure 3A. When one outlying data point (salivary cortisol concentration of 48 nmol/L with corresponding total plasma cortisol of 387 nmol/L, shown by an arrow in Figure 3A) was removed from the analysis, correlation between plasma and salivary cortisol concentrations was closer (*R* = 0.73, *P* <0.0001). The correlation between endogenous total plasma cortisol and urinary cortisol:creatinine ratio levels (*R* = 0.56, *P* <0.0001) is shown in Figure 3B. When one outlying data point was removed (shown by an arrow in Figure 3B), the plasma-urinary cortisol relationship was closer (*R* = 0.65, *P* <0.0001).

In subjects given oral hydrocortisone, the correlation between total plasma and salivary cortisol concentrations (*R* = 0.83, *P* <0.0001, Figure 3C) was stronger than endogenous cortisol data, whereas plasma-urine cortisol relationship was similar (*R* = 0.61, *P* <0.0001, Figure 3D), compared to the endogenous data.

In subjects given intravenous hydrocortisone, there was a high correlation was between total plasma and salivary cortisol (*R* = 0.94, *P* <0.0001, Figure 3E), and between total plasma cortisol and urinary cortisol:creatinine ratio (*R* = 0.82, *P* <0.0001, Figure 3F). The relationship between plasma and salivary or urinary cortisol was non-linear.

The correlation between plasma free cortisol and salivary or urinary cortisol was similar (data not shown) to that between total plasma cortisol and the noninvasive measurements (Figure 3).

## Discussion

This study investigated dexamethasone-suppressed normal volunteers as a model of adrenal insufficiency, which also allows direct comparison with the participants’ own endogenous cortisol concentrations in plasma, saliva and urine as a control. In doing so, the baseline post-dexamethasone 0830-0900 h cortisol was low, as it is in patients with adrenal insufficiency prior to taking their morning medication. Endogenous cortisol levels peak approximately half an hour after waking, and we have compared like time points rather than the maximum endogenous cortisol versus the peak following exogenous hydrocortisone administration. The morning 0900 h cortisol is a common clinical test of hypothalamic-pituitary-adrenal axis function, but since this is not standardized to waking time, is not necessarily the highest concentration of the day.

We examined cortisol concentrations following a morning dose of hydrocortisone 20 mg which is the standard tablet size available in Australia. Despite evidence of increased mortality with hydrocortisone doses above 25–30 mg per day [9, 11], this dose is still widely used in clinical practice. After the administration of hydrocortisone 20 mg, cortisol concentrations in plasma, saliva and urine samples were significantly supraphysiological. Most of the 4-hour total plasma cortisol levels following 20 mg oral hydrocortisone were above the 90^th^ centile shown in the normogram proposed by Mah *et al.*[21] for individual adjustment of hydrocortisone dosage.

We have investigated the utility of measuring cortisol in saliva and urine, examining the potential for non-invasive sampling as a means to monitor glucocorticoid dosage. Furthermore, saliva and urine cortisol may more closely reflect plasma free cortisol concentrations. The higher *R* values observed in the correlations between total plasma and saliva/urine cortisol measures after oral and intravenous hydrocortisone compared to on the endogenous control day likely reflect the higher proportion of plasma free cortisol under the pharmacological conditions. Under normal physiological conditions, approximately 90% of total plasma cortisol is protein bound, mainly to CBG. Saturation of CBG occurs at total plasma cortisol concentration of 450–550 nmol/L [33, 34], therefore even the 20 mg oral hydrocortisone dose readily saturates CBG binding capacity, leading to large increases in free plasma, saliva and particularly urine cortisol. This effect was more pronounced after the intravenous 50 mg dose. We also demonstrated that the clearance of cortisol was significantly higher after the intravenous dose, suggesting that clearance (a combination of hepatic metabolism and urinary excretion) is proportional to the peak concentration achieved. By 2 hours, the plasma cortisol concentrations were similar between the oral 20 mg and intravenous 50 mg dose, demonstrating that most of the extra dose is cleared rapidly. It is unknown however, to what extent the higher peak cortisol concentrations observed during the “stress” dose lead to a greater or longer-lasting degree of glucocorticoid action intracellularly. Furthermore, there is recent evidence that 11 beta hydroxysteroid dehydrogenase type 1 (11βHSD1) is a crucial mediator of the clinical effects of glucocorticoid excess, suggesting the importance of regeneration of active cortisol from cortisone at tissue level [35]. It cannot be assumed therefore, that the supraphysiological free cortisol is effectively cleared without causing significant tissue effect.

The elevated cortisol concentrations and high urinary excretion measured in our study after a morning dose of hydrocortisone 20 mg reinforces the concept that the traditional hydrocortisone replacement regimen of 30 mg daily is excessive in most patients with adrenal insufficiency [7]. In the absence of a reliable biomarker for glucocorticoid activity, clinical assessment of glucocorticoid replacement therapy has been suggested as the method of choice for dose adjustment [10, 22]. The use of cortisol day curves has been used in patients who are not feeling well despite clinical adjustment of hydrocortisone doses [7], but these are labour and time intensive, requiring repeated blood sampling as a day admission. We found that the measurement of a single plasma cortisol at 2 h gives a good indication of the peak cortisol concentration, while Mah *et al.*[21] have shown that a plasma cortisol level at 4 hours correlates well with the cortisol AUC.

In assessing the noninvasive methods (saliva and urine samples), we found significantly positive correlations with total plasma cortisol. Previous studies examining the utility of salivary cortisol as a monitoring tool have yielded conflicting results [18, 26]. In the latter study [18], salivary cortisol levels in hospital inpatients might be relatively higher than in healthy outpatients recruited in our study which may explain the difference in study outcomes. Similar to our study, Lovas *et al.*[27] reported a high correlation between plasma and salivary cortisol concentrations (*R* ≥0.83, *P* <0.002) after oral intake of cortisone acetate and concluded that salivary cortisol measurements can be used for monitoring of glucocorticoid replacement therapy. Salivary cortisol day curves have been shown to be useful in the individual adjustment of glucocorticoid replacement therapy in patients with Addison’s disease to avoid over-replacement, particularly in the afternoon and evening, with subsequent reduction in sleep disturbances [36]. However, there are known limitations of salivary cortisol sampling. Twenty five percent of our subjects showed evidence of contamination by residual oral hydrocortisone (particularly at the 1 h sample time point) which has been reported previously [1, 26]. Therefore, we would advise the measurement of salivary cortisol at 2 h (which corresponds closely to the measured T_max_) to assess peak concentration if this method is used for monitoring of hydrocortisone replacement therapy, but advise caution in interpreting very high salivary cortisol results. Salivary cortisol may also have limitations in patients with other co-morbidities or on multiple medications. The use of spot urinary cortisol:creatinine ratios may prove to be useful under certain circumstances, especially given the relative ease of collection and the fact that this measure was stable across a one hour period between 2 and 3 hours after hydrocortisone administration. Further research is necessary using the recommended hydrocortisone morning dose of 0.12 mg/kg [21] to define treatment reference range targets for post-dose salivary cortisol and urinary cortisol:creatinine ratio before either measure assumes clinical utility.

Intravenous hydrocortisone 50 mg achieved very high 30 minute cortisol levels, given the true peak level would be achieved immediately after the intravenous bolus. The mean 30 minute total plasma cortisol of 2450 nmol/L was well above the levels reported in severe stress, such as septic shock (mean, 880 nmol/L) [37] or following coronary artery bypass surgery (median, 744 nmol/L) [38]. In patients with adrenal insufficiency given intravenous boluses of hydrocortisone 50 mg 6-hourly, peak plasma cortisol levels were over 100 μg/dL (2760 nmol/L), and nadir levels remained elevated at 40–50 μg/dL (1100–1380 nmol/L) [39]. It was recommended that the dose of hydrocortisone should not exceed 200 mg/day given as a continuous infusion or as intravenous boluses 4–6 hourly [39]. Total plasma cortisol was greater than 400 nmol/L at 5 h in 88% of subjects in the intravenous hydrocortisone group, suggesting that if using intermittent injections, a 6-hourly interval would be adequate for most patients with adrenal insufficiency requiring glucocorticoid supplementation therapy during acute stress, given that the tissue half-life of cortisol is longer than its plasma half-life (8–12 hours *vs*. 66 minutes, respectively [40]). In a minority of patients, an increased frequency of administration may be required; the monitoring of trough plasma levels at 5-6 h could identify such patients, particularly if they are not clinically responding to stress doses of hydrocortisone. The problem of high peaks and low troughs is overcome using a continuous infusion of intravenous hydrocortisone as recently demonstrated by Taylor *et al.*[41]*.* They have shown that infusing hydrocortisone 200 mg/24 h produces a steady state cortisol concentration with mean concentration of 835 nmol/L.

The peak cortisol, AUC_0–5_ and T_max_ values were variable after oral hydrocortisone, but on average, T_max_ occurred at around two hours following the dose. Previous studies have reported on the inter-individual variability in the plasma cortisol pharmacokinetics after fixed doses of oral cortisone acetate [20] or hydrocortisone [21]. Causes of variation include differences in body weight [21], gastrointestinal absorption and hepatic first-pass metabolism of hydrocortisone [42], and hepatic 11β-hydroxysteroid dehydrogenase type 1 activity in regards to cortisone acetate.

Dexamethasone was used to suppress endogenous cortisol production, a procedure which has been used previously for the evaluation of cortisol pharmacokinetics following administration of hydrocortisone [14, 33]. We used a higher dose of dexamethasone (4 mg as opposed to 1 mg) to ensure complete suppression throughout the study day. This dose of dexamethasone invariably maintains plasma cortisol concentrations below 50 nmol/L until at least 1600 h in normal volunteers (W Inder, unpublished observation). Dexamethasone did not result in a significant effect on CBG concentrations compared to the endogenous control day, and we could find no literature to suggest that acute glucocorticoid administration affects CBG concentrations.

We were not able to undertake complex cortisol pharmacokinetic modeling or measure cortisol levels over 24 hours which would require inpatient admission. We did not have access to an assay for direct measurement of plasma free cortisol (and this is not readily available in clinical practice). However given the study was carried out in normal volunteers, Coolens’ equation [25] is an accepted surrogate measure of plasma free cortisol, which has been used in a recent study in the assessment of hydrocortisone replacement [43]. While Barlow *et al.*[44] found that Coolens’ equation underestimated measured cortisol, Ho *et al.*[37] reported close agreement between measured and calculated free cortisol. The number of participants was relatively small and did not allow further analysis of other factors which may affect clearance such as body mass index.

## Conclusions

We confirm that a morning dose of hydrocortisone 20 mg is supraphysiological and excessive for routine maintenance. It results in very high free cortisol concentrations which lead to increased urinary cortisol excretion. The measurement of plasma, salivary or urinary cortisol at 2 h after an oral dose gives a good indication of peak cortisol concentrations, but further research defining optimal targets for salivary cortisol and urinary cortisol:creatinine ratio following true physiological hydrocortisone replacement are required before recommending these measures in clinical practice. An intravenous bolus of hydrocortisone 50 mg achieved very high cortisol levels within 30 minutes of administration. The routine biochemical monitoring of stress glucocorticoid supplementation therapy is not recommended, but the measurement of trough levels at 5-6 h may identify a minority of patients who require more frequent intravenous dosing.
